# Refractive index sensing and surface-enhanced Raman spectroscopy using silver–gold layered bimetallic plasmonic crystals

**DOI:** 10.3762/bjnano.8.249

**Published:** 2017-11-24

**Authors:** Somi Kang, Sean E Lehman, Matthew V Schulmerich, An-Phong Le, Tae-woo Lee, Stephen K Gray, Rohit Bhargava, Ralph G Nuzzo

**Affiliations:** 1 Department of Materials Science and Engineering, University of Illinois at Urbana-Champaign, Urbana, Illinois 61801, USA; 2 Department of Chemistry, University of Illinois at Urbana-Champaign, Urbana, Illinois 61801, USA; 3 Beckman Institute for Advanced Science and Technology, University of Illinois at Urbana-Champaign, Urbana, Illinois 61801, USA,; 4 Chemistry Division and Center for Nanoscale Materials, Argonne National Laboratory, 9700 Cass Ave., Lemont, Illinois 60439, USA

**Keywords:** finite-difference time-domain, nanoimprint soft lithography, plasmonics, surface plasmon resonance

## Abstract

Herein we describe the fabrication and characterization of Ag and Au bimetallic plasmonic crystals as a system that exhibits improved capabilities for quantitative, bulk refractive index (RI) sensing and surface-enhanced Raman spectroscopy (SERS) as compared to monometallic plasmonic crystals of similar form. The sensing optics, which are bimetallic plasmonic crystals consisting of sequential nanoscale layers of Ag coated by Au, are chemically stable and useful for quantitative, multispectral, refractive index and spectroscopic chemical sensing. Compared to previously reported homometallic devices, the results presented herein illustrate improvements in performance that stem from the distinctive plasmonic features and strong localized electric fields produced by the Ag and Au layers, which are optimized in terms of metal thickness and geometric features. Finite-difference time-domain (FDTD) simulations theoretically verify the nature of the multimode plasmonic resonances generated by the devices and allow for a better understanding of the enhancements in multispectral refractive index and SERS-based sensing. Taken together, these results demonstrate a robust and potentially useful new platform for chemical/spectroscopic sensing.

## Introduction

Studies of surface plasmons have attracted significant attention due to the diverse range of applications and processes in which they can be exploited. These applications include, but are not limited to: laser emission, light trapping, optical modulation, and label-free means of chemical or biological sensing [1–6]. Surface plasmons are collective oscillations of conduction electrons near metal surfaces that are excited by electromagnetic radiation incident at a metal/dielectric interface. This results in an evanescent decaying electric field that extends from the metal surface for ≈100–200 nm (surface plasmon polaritons), or it can also manifest as a localized surface plasmon resonance at the surface of a metal nanostructure (localized surface plasmons). The attributes of these excitations are highly sensitive to local refractive index changes, which in turn allow for their exploitation in chemical and biological sensing [7–9]. In this way, surface plasmonic resonance (SPR) sensors are promising as an analytical technique for real-time, fully label-free detection of molecules, both quantitatively and qualitatively, as well as for monitoring surface interactions [5,10–11]. Surface-enhanced Raman spectroscopy, better known as SERS, is another important analytical application that utilizes enhanced electromagnetic fields generated by surface plasmons [12–13]. Raman scattering signals can be dramatically amplified on a plasmonic substrate, reportedly by as much as 10 to 11 orders of magnitude, reaching levels of sensitivity suitable for single molecule detection [14–15].

In general, photons cannot directly elicit plasmonic excitations on metal films in air due to conservation requirements [16–17]. To compensate for the mismatch in momentum between an incident photon and a plasmonic resonance, most studies to date have focused on metallic nanostructures such as nanoparticles, line gratings, and nanoscale holes or voids to effect couplings and further obtain stronger electromagnetic fields and higher spatial resolution from localized surface plasmon resonance (LSPR) [18–27]. Many fabrication methods have been described that provide structures capable of generating these plasmonic features [28–33]. Our work in this area has exploited soft nanoimprint lithography, a technique that permits reproducible replication of precisely defined nanometer-sized features over a large area (greater than 1 × 1 cm^2^), as a way to fabricate quantitative imaging-mode and multispectral plasmonic optics [23–24,34–36]. This fabrication method yields highly uniform arrays of nanoholes in a dielectric substrate that upon metallization provide a plasmonic platform for SPR sensing and SERS [22–26,37–38].

Noble metals such as Au and Ag are the most commonly used plasmonic materials because they generate strong plasmonic resonances at visible and near-infrared frequencies [39]. Metallic Ag generates a stronger evanescent field and a narrower plasmon resonance than Au, which in principle is advantageous for both optical sensing and SERS [40–42]. It is Au, however, that is more widely utilized for analytical applications due to its long-term environmental stability, ease of surface modification, and biocompatibility [43]. The present study explores means for realizing synergy in utilizing the complementary attributes of each material. Here we examine a bilayer/multi-metallic Ag/Au plasmonic crystal (PC) motif that is characterized by higher analytical sensitivity and more strongly enhanced electric fields than our previously reported monometallic PC systems. Manipulating the composition of the thin metal films, their spatial distribution, and the design rules of the PC optical elements (in terms of the lattice parameter and the size of the features of the nanoholes) is found to be an effective approach to optimizing the response in multispectral and SERS-based sensing. To illustrate the properties of these devices, the optical response from exemplary PCs was acquired by 0^th^-order transmission measurements. Finite-difference time-domain (FDTD) calculations were performed to assist in characterizing how each system behaved in order to understand and obtain an optimized device form factor. The data illustrate that Ag/Au bimetallic PCs possess potential for implementation in chemical sensing.

## Experimental

### Materials

The reagents were used as received without further purification unless otherwise specified. Spin-on-glass (SOG) 315F was purchased from Filmtronics and was filtered twice sequentially using 0.22 μm (Millipore) and 0.02 μm (Whatman Anotop 10) syringe filters immediately before use in order to remove nanoparticles in the SOG sol formed by hydrolysis [26]. Polydimethylsiloxane (soft PDMS; Sylgard 184, Dow corning) was prepared in a 10:1 ratio of PDMS base with curing agent. Hard PDMS components, poly(25–30% methylhydrosiloxane)-(dimethylsiloxane) (HMS-301), poly(7–8% vinylmethylsiloxane)-(dimethylsiloxane), (VDT-731), platinum divinyltetramethyldisiloxane (SIP6831.1) and (1,3,5,7-tetravinyl-1,3,5,7-tetramethylcyclotetrasiloxane) (7900), were purchased from Gelest. Polyethylene glycol (PEG, *M*_W_ = 10,000 g/mol) and benzenethiol (BT) (99.99 %) were purchased from Aldrich. 100% ethyl alcohol (Decon) was used to solvate benzenethiol. Ultrapure water (18.2 MΩ·cm) was generated using a Millipore Milli-Q Academic A-10 system and used to prepare the PEG buffer (0–5.6 wt %) solutions.

### Plasmonic crystal fabrication via soft nanoimprint lithography

**Figure 1:**
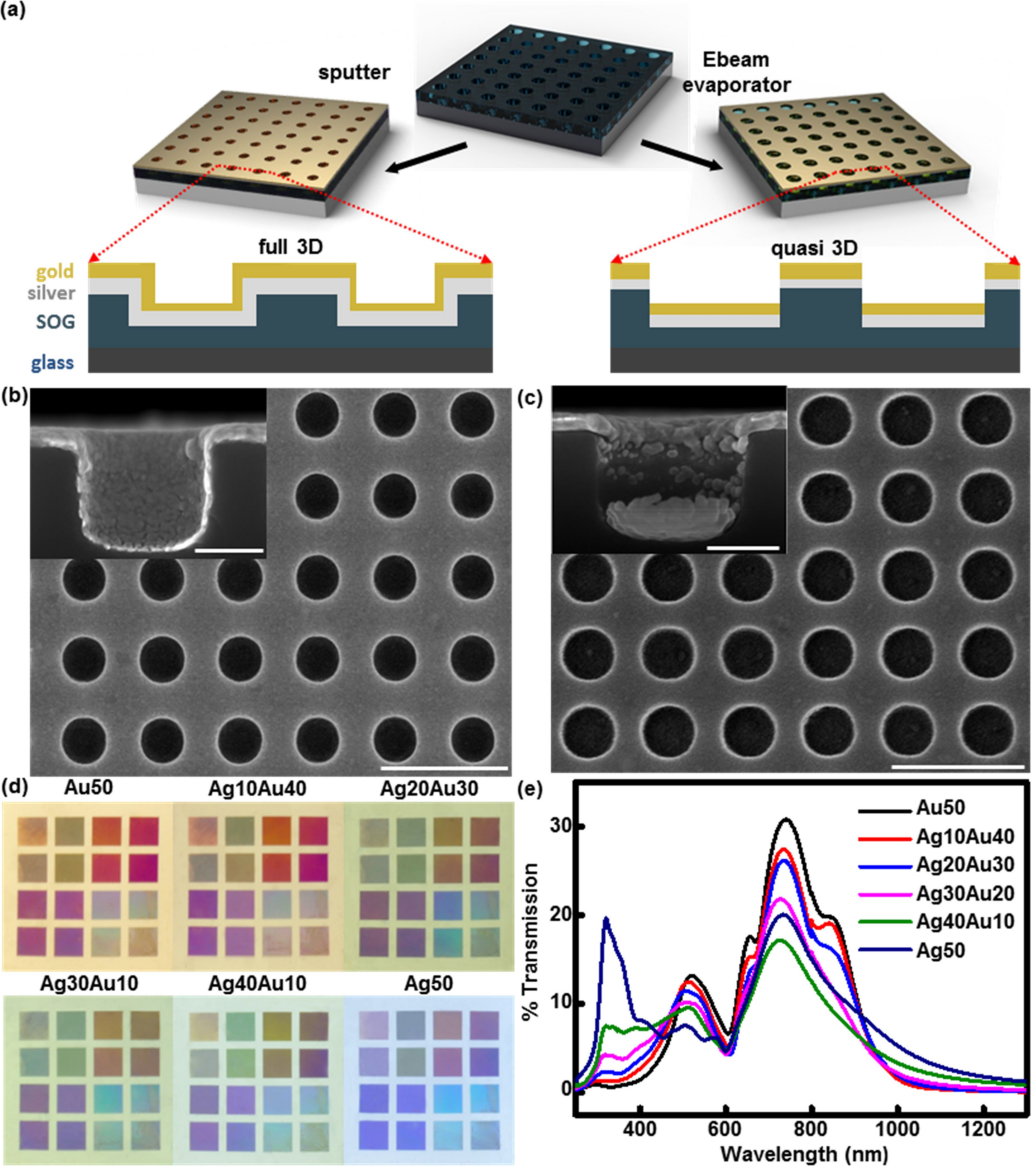
(a) 3D and 2D schematic illustration of the full-3D and quasi-3D plasmonic crystals (PCs). To fabricate the metal films, sputtering was used for the full-3D PC and e-beam evaporating was used for the quasi-3D PC. SEM images of a (b) full-3D and (c) quasi-3D PC with diameters and periodicities of ≈380 and 580 nm. The scale bar corresponds to 5 μm. Inset: cross-sectional SEM image of an individual nanohole. The scale bar corresponds to 100 nm. (d) Optical images of embossed PCs with different metal layer compositions: ≈50 nm Au (Au50), ≈10 nm Ag/≈40 nm Au (Ag10Au40), ≈20 nm Ag/≈30 nm Au (Ag20Au30), ≈30 nm Ag/≈20 nm Au (Ag30Au20), ≈40 nm Ag/≈10 nm Au (Ag40Au10), ≈50 nm Ag (Ag50). The hole depth is ≈340 nm and the hole spacing varies from ≈500 nm to ≈1740 nm. (e) Normal incident transmission spectra of five full-3D PCs with different metal composition.

The PCs were fabricated using soft nanoimprint lithography as previously reported [22–26,37–38]. In brief, a composite hard-PDMS/soft-PDMS mixture was cast on a patterned photoresist master with arrays of nanohole relief structures in order to fabricate the PCs. A glass slide was fully covered with liquid SOG, and spin cast (≈950 rpm for 6 s) to produce a thin, uniform layer of liquid SOG on the glass slide surface. The PDMS stamp was then pressed into the SOG-coated glass slide and fastened in place to achieve conformal contact between the PDMS stamp and SOG film. Before baking, the sample was left at room temperature for 7 min to facilitate evaporation of volatile organic components of the SOG material. The sample was soft baked at 110 °C for 5 min, followed by the careful removal the PDMS stamp. The embossed SOG film was then further cured at 200 °C for 5 min followed by baking at 160 °C overnight. Finally, the fully cured SOG film was annealed at 450 °C under nitrogen for 1 h. The replicated SOG nanostructures consist of well-defined square arrays of nanoholes, patterned using sixteen different design rules that offer a broad variation of response to optical frequencies. A ≈5 nm titanium dioxide adhesion layer was deposited using atomic layer deposition (Cambridge nanotech) on the embossed SOG nanostructure followed by deposition of a ≈50 nm metallic film (Au, Ag or both) via one of the various methods. Sputter deposition in a 5 mTorr argon atmosphere (AJA International) was utilized for substrates prepared for bulk refractive index (bulk RI) sensing experiments and electron beam (e-beam) evaporation (Temescal) was used for samples made for SERS measurement. The schematic illustration of both SOG PC structures is given in Figure 1a and the scanning electron microscopy (SEM) images of nanohole arrays of different metal distributions are shown in Figure 1b (sputter deposition) and Figure 1c (e-beam evaporation). The island-like metallic film structures of the quasi-3D structures (shown in inset of Figure 1c) were found to be susceptible over time to the penetration of liquid solution into the interfaces formed between the metal films and the SOG substrate. To prevent the degradation in performance that this engendered, a conformal ≈6 nm thick Al_2_O_3_ passivation film was deposited on top of the metal by atomic layer deposition (Cambridge Nanotech).

### Bulk refractive index sensing via transmission-mode spectroscopy

Transmission spectra in air and bulk RI dependent PC data were measured using a Varian 5G UV–vis–NIR spectrophotometer with normal incident light and no temperature control. Bulk RI measurements were carried out using methods that have been previously reported [22,24,26,37,44–45]. In this protocol, a PDMS flow cell was mounted on top of the PC and PEG buffer solutions of increasing concentration (pure water, 1.4, 2.8, 4.8, and 5.6 wt %) were injected into the PDMS flow cell with a syringe pump (Harvard Apparatus) at a flow rate of 0.1 mL/min. To change the solution, a new PEG solution was injected at a flow rate of 1.0 mL/min at the beginning stage for two minutes to completely flush the previous solution from the PDMS cell. This was followed by injection at a normal flow rate of 0.1 mL/min. Transmission spectra over a wavelength range of 355–1000 nm were collected throughout the process in order to monitor changes in multiple plasmonic responses to changes in the surrounding dielectric environment. The refractive index of the PEG solutions was measured using an Abbe refractometer, corrected to 25 °C.

### Integrated multispectral response calculation

In quantitative work, we consider both the position and intensity changes that occur in the plasmonic features present in the transmission spectrum over the entire range of collected wavelengths. The absolute difference values were calculated using the differences between transmission spectra collected in each PEG solution and a spectrum recorded in pure water. These values were integrated to account for wavelength dependence of both negative and positive changes in spectroscopic intensity using Equation 1, a value having units of Δ%T·nm.

[1]
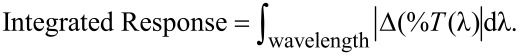


### Surface-enhanced Raman spectroscopy (SERS) measurements

A self-assembled monolayer of benzenethiol on top of the quasi-3D PCs was prepared by immersing them in a 15 mM benzenethiol ethanolic solution for 12 h, rinsing thoroughly with ethanol, and then drying with a stream of nitrogen gas. SERS measurements were made using a SENTERRA dispersive Raman microscope (Bruker Optics) with an excitation laser wavelength of 785 nm, an excitation power of ≈5 mW, focal length of 45 mm and acquisition time of 30 s. Raman spectra were collected over a Raman shift range of 500–1800 cm^−1^.

### Finite-difference time-domain simulation of plasmonic nanostructures

A set of 3D FDTD simulations were used to model the normal incidence transmission spectra in air and water and the electromagnetic field distribution for full-3D PCs. The unit cell geometry was defined as an infinite square array of nanostructured holes on a metal film that are parallel to the *x*–*y* plane with a semi-infinite SOG material under the nanoholes and air above them. The unit cell spacing was 2 nm in all three dimensions with a total unit cell size of *N_x_* × *N_y_* × *N_z_* = 292 × 292 × 1200 grid points. The total simulation time for each unit cell was 100 fs. Perfectly matched uniaxial layers were applied on both sides of the z-grid to avoid artificial reflection errors from the domain boundaries. Appropriate periodic boundary conditions were used to define the square array. The frequency-dependent Au^22^ and Ag^46^ permittivities are described by the Drude–Lorentzian model over a wavelength range of 355–1500 nm. The dielectric constants for SOG and air were taken to be 1.43 and 1.00, respectively.

## Results and Discussion

### Structure and properties of bimetallic plasmonic crystals

Periodic nanostructures in metal films have been widely studied as a system to better understand the underlying physics of plasmon resonances as well as to develop high-performance platforms for a variety of SPR applications [21,46–47]. Compared to other work on nanoparticles or 2D arrays, the 3D plasmonic constructs described here have notable differences in terms of both the underlying physics as well as the analytical approaches they support. A specific contrast compared to freely diffusing nanoparticles in solution, sensing on plasmonic crystals provides specific advantages, being well-suited for use as chemoresponsive/multispectral imaging optics as well as not being subject to temporal instabilities that arise from interparticle interactions mediated via operando sensitive attributes of surface charge. This intrinsically makes plasmonic crystals more attractive for sensing applications as the need to modify the surface chemistry/charge figures heavily in design of capacities to support the recognition of specific solution-phase analytes. Additionally, local electromagnetic hotspots and fano resonances generated at the surface are much more accessible by design in photonic crystal systems due to the intrinsically controllable geometry of the array openings. In contrast, the interaction of nanoparticles can be used to modulate the effective electromagnetic field generated at hotspots but these ultimately depend on solution conditions in ways that can be hard to control and vary both within and between assays. The optimal morphology for a given application will necessarily depend on the specific parameters required including response time, harshness of solution conditions, and overall spectroscopic detection figures-of-merit required. The work described herein demonstrates how a systematically varied metallic composition can be used to tailor the bulk RI and spectroscopic sensing capabilities of PC optics. The work establishes figures-of-merit for these tailored systems and might serve to benefit analysis in biological as well as other, more general, analytical contexts. This work further establishes protocols that afford effective materials approaches to stabilize Au/Ag metal multilayer PC optics against environments that would otherwise degrade their performance in use.

Efforts in this field have been directed particularly toward the unique optical properties of periodic nanostructures because of their capacities for extraordinary optical transmission, visual wavelength responsiveness, and high optical sensitivity to changes in the local environment [48–50]. In this study, we used square arrays of nanoholes molded into the surface of an inorganic SOG film using soft nanoimprint lithography. The SOG-supported PC structure affords a chemically and thermally stable sub-wavelength optical device, especially when a protective metal oxide overlayer is included, for SPR sensing and for SERS measurements, as has been discussed in previously published work [26].

The SPRs produced using nanostructured metal films can be easily tuned by adjusting the geometric shape, thickness, and composition of the metal film, as well as the surrounding dielectric environment. Sputter coating and e-beam evaporation are both widely used tools for fabricating metal thin films, yet the distribution of the deposited metal film resulting from each of the two methods are quite different due to the distinctive deposition characteristics. The step coverage of e-beam evaporation, in particular, is poor since it only offers an essentially unidirectional collision of source material atoms with the substrate, with most of the metal being deposited on the surface and bottom of the nanoholes. Sputtering, in contrast, is a less-directed deposition process that conformally coats the entire substrate. We have termed the metal nanostructures fabricated by e-beam deposition as quasi-3D structures because the metal disk at the bottom of the nanohole is physically separated from the metal film on the top of the SOG substrate, while the PCs with more continuous/conformal metal layers formed by sputter deposition are termed full-3D PCs [37]. Figure 1a schematically illustrates the metal film distribution differences resulting from the fabrication using these two methods. The geometric differences in the metal films of the quasi-3D and the full-3D PCs were characterized using cross-sectional SEM imaging (inset of Figure 1b,c). The top view SEM images of PCs in Figure 1b,c demonstrate that PCs consist of uniform square arrays of nanoholes with a periodicity of ≈580 nm. Despite the fact that the diameter of the embossed SOG nanostructures of both PCs are identical (≈380 nm), the final diameter of nanoholes in the full-3D PC (Figure 1b) appears slightly smaller than those of the quasi-3D PC (Figure 1c). This effect was attributed to differences in sidewall metal deposition resulting from the specific technique used.

In addition to changing the geometry of the nanohole arrays and the metal by altering the metal film deposition method, modification of the metal film composition provides an additional means through which the optical response of the PC can be systematically controlled. In this study, we used bimetallic layers to engineer the plasmonic response of the device over a wider wavelength range, here exploiting the unique plasmonic modes of Ag in the visible range to supplement and enhance those provided by Au. This distinctive PC design further serves to compensate for the chemical instability of Ag by coating it with thin films of Au. To explore the variation in function resulting from the double layer film structure, we used five different ratios of Ag and Au while maintaining a constant overall metal film thickness on the SOG nanohole arrays. The ratios used were: 50 nm Au (Au50), 10 nm Ag/40 nm Au (Ag10Au40), 20 nm Ag/30 nm Au (Ag20Au30), 30 nm Ag/20 nm Au (Ag30Au20), and 40 nm Ag/10 nm Au (Ag40Au10). Figure 1d presents optical images of an entire series of PCs with varying compositions of Ag and Au. The sixteen squares (4 × 4 mm^2^) given on each plasmonic substrate exhibit distinctive colors due to light scattering from the nanohole arrays according to the different design rules, with periodicities ranging from 0.49 to 1.75 μm and corresponding hole diameters ranging from 0.17 to 1.12 μm. The color changes seen, from the typical color of bulk Au to that of Ag, follow an intuitive trend as the ratio of Ag in the metal film increases. Metrological data developed using SEM demonstrated a high fidelity of the replicated structure to its imprint master, as illustrated in the nearly identical geometry (≈340 nm hole depth, ≈380 nm hole diameter, and ≈580 nm hole spacing) of an exemplary pair of PCs shown in Figure 1b (full-3D) and Figure 1c (quasi-3D). The former structures have a particularly useful response in the visible wavelength region of the optical spectrum. This is illustrated by the data presented in Figure 1e, which shows the 0^th^-order transmission spectra for six full-3D PCs recorded in air for varying ratios of Au and Ag. The variations in the transmission spectra can be attributed to the changes in the film composition (Ag and Au thickness). The small discontinuity seen near 800 nm is an instrumental artifact, which results from a change of detectors during the scan. In Figure 1e, the largest transmission peak, spanning the near-infrared (NIR) range, is blue-shifted and its intensity decreases as the proportion of Ag in the metal film increases. Across the near UV–vis regions, enhanced transmission magnitudes and more spectroscopic features are observed as the mass-coverage of the Ag increases. The intensity of the characteristic peak for the Au surface plasmon resonance (at ≈500 nm) is reduced as the proportion of Ag increases, while the peak intensity (at ≈350 nm) corresponding to the bulk plasmon mode of Ag is enhanced. These qualitative trends are more quantitatively described by the results of theoretical modeling.

### Finite-difference time-domain modeling

FDTD calculations provide theoretical understanding of how the electromagnetic field interacts with the PC [51–53]. The transmission spectra of the periodic nanohole arrays in each of the PCs have multiple optical responses, such as localized surface plasmon resonances (LSPRs), Bloch wave surface plasmon polaritons (BW-SPPs), Wood’s anomalies (WAs), or a combination of these features [28,48,54–60]. Contributions from the transmission of light also originate from the coupling of light directly transmitted through the metal film, as well as from the background metal absorption (at ≈500 nm for Au and ≈350 nm for Ag). The peaks in the transmission spectra, which correspond to BW-SPPs and WAs, are correlated with the periodic structure of the nanoholes arrays, whereas LSPRs can be created by specific sub-wavelength-sized features of the metallic structures.

**Figure 2:**
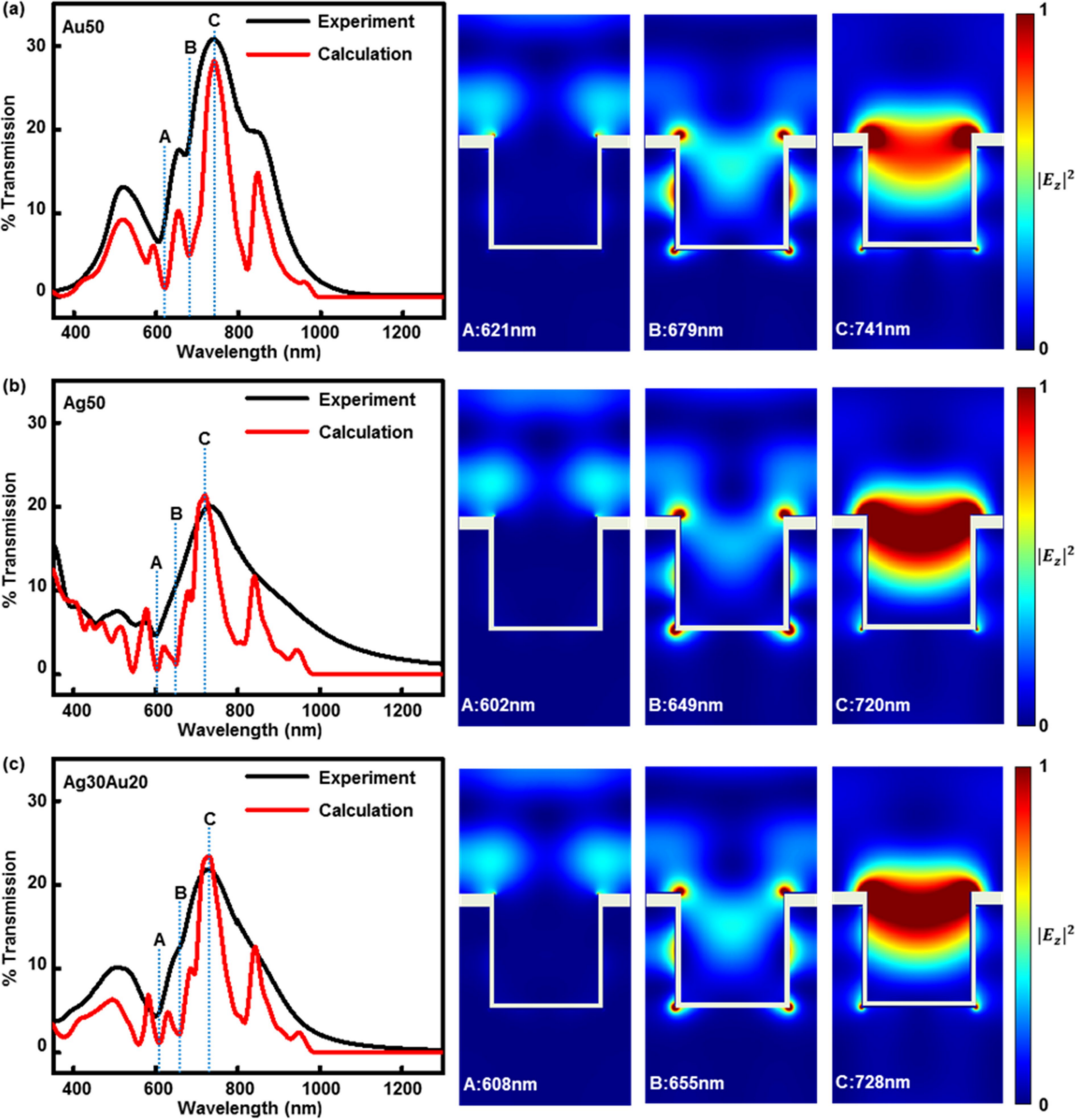
Experimental transmission spectra (black), electrodynamic modeling transmission spectra (red), and 2D calculated electric field plots corresponding to wavelengths for peaks labeled as A, B, and C in the transmission plot of (a) Au50, (b) Ag50 and (c) Au20Ag30 full-3D PCs (≈380 nm hole diameter, ≈340 nm relief depth, ≈580 nm hole spacing, ≈50 nm top metal layer, ≈20 nm metal sidewall, and ≈15 nm bottom metal layer).

The appropriate optical constants for each material and the geometrical model of the periodic nanohole array are crucial for FDTD modeling because all of the plasmonic modes are highly sensitive to the structural details and dielectric properties of the environment. The design rules for an optimized PC were used in the calculations (see below), and based on experimentally measured data, they were defined as a square array of nanoholes in a layer of SOG with a diameter of 380 nm, depth of 340 nm, and a center-to-center spacing of 580 nm. The dimensions of the metal film above the SOG nanohole arrays were as follows: top metal layer of ≈50 nm thickness, bottom metal layer of ≈20 nm thickness, and sidewall metal layer thickness of ≈15 nm (details are given in Supporting Information File 1, Figure S1). The different thickness values account for the shadowing effect which limits the degree of conformal coverage realized in the sputter coating metallization step. In the calculation, we used a Drude plus two-pole Lorentzian model to obtain the dielectric constant of the metal as a function of wavelength [48,61]. Figure 2 presents the experimentally measured and calculated normal incidence transmission spectra and electric field distributions around the nanoholes for full-3D PCs with Au50 (Figure 2a), Ag50 (Figure 2b), and Ag30Au20 (Figure 2c) mass-coverage metal films. The Al_2_O_3_ passivation layer was not included in the calculations as it was found to impart only very small modifications to the experimentally determined transmission spectra.

The calculations and experimental data shown are for PCs in air, and assignments for specific features seen in the data are revealed by the calculations. The largest peak (labeled C in the spectra) is the result of strong LSPR excitations that are confined in the nanohole. Fano-like resonances, appearing as peak minima or maxima, are correlated with either BW-SPPs or WAs [22,60]. The wavelengths of the expected BW-SPPs can be predicted using Equation S1 in Supporting Information File 1 and solutions for this equation are listed in Supporting Information File 1, Table S1. From this theoretical analysis, we can verify that the peak minima labeled A in the transmission spectra originate from BW-SPPs. At these wavelengths, the field intensity distributions seen near the interface of the top metal layer and air stand in good agreement with the characteristic field distribution expected for BW-SPPs. Even so, many of the optical features observed in the transmission spectra are likely the result of complex interactions of light diffraction and concurrent plasmonic modes. The electric field distributions calculated for the wavelength at position B in each of the spectra are examples of features involving such coupling of multiple plasmonic modes. The intensity concentrated in the nanohole and near the sidewalls, for example, likely corresponds to LSPRs. Moreover, electric fields located at the metal–air interface are largely due to BW-SPPs and/or WAs.

The FDTD results (Figure 2) are consistent with the experimental results in terms of the dependence of the 0^th^-order transmission spectra as a function of changes in the metal overlayer composition (presented in Figure 1e). The data show, for example, that the peak intensities are reduced and the intensity of the transmission maxima (position C) are blue-shifted with increasing Ag content. The calculations further demonstrate several BW-SPP features that characterize transmission for the Ag PCs at wavelengths around 400 nm – a complexity in the blue wavelength region expected for Ag in contrast to Au. The optical features appearing between 400–600 nm for the Ag PCs suggest a possibility for a highly sensitive response for such PCs at near-UV and visible wavelengths [62–63].

### Bulk refractive index sensitivity of bimetallic plasmonic crystals

A single resonance peak analysis methodology does not provide a suitable means to determine the RI sensitivity of an imaging sensor. For this reason, we employed a multispectral protocol to quantify figures-of-merit (FOM) for sensitivity, as described in earlier reports on chemical sensing using PCs as an optical element for imaging and spectroscopic detection [22,24,26,37]. Here we used a flow cell design, the same as that previously reported, to expose the PC to PEG solutions of varying concentrations to determine a FOM for its multispectral RI sensitivity. Because sensitivity to bulk RI changes has been previously shown to be better for the full-3D PC structure as compared with the quasi-3D counterpart, the transmittance spectra for the former over a range of 355–1000 nm were collected as a function of time while PEG solutions of different concentration (from 1.4 to 5.6 wt %) were passed through the flow cell [37]. Integrated multispectral RI responses of five full-3D PCs with different mass-coverage metal films were measured to investigate and validate the best design for the PC for bulk RI sensing.

To determine an optimal nanohole array design for RI sensing in the visible wavelength range before considering the effects of mass fraction in metal thin film on plasmon resonances, we performed bulk RI sensing measurements (Supporting Information File 1, Figure S2a) and FDTD calculations (Supporting Information File 1, Figure S2b) using four design rules of nanoholes chosen from sixteen squares on a PC: 580 nm (p580), 780 nm (p780), 1100 nm (p1100), and 1600 nm (p1600). From the results of bulk solution-phase RI sensing (shown in Supporting Information File 1, Figure S2a), the most sensitive geometry for RI changes measured over a wavelength range of 300–800 nm was found to be a nanohole array with ≈580 nm periodicity and ≈380 nm hole diameter. The FDTD calculation for this structure (presented in Supporting Information File 1, Figure S2b) confirms the physical origin of the bulk RI sensing results in which the p580 Au50 full-3D plasmonic substrates generated the strongest plasmonic features at frequencies in the visible range (wavelengths spanning 600–800 nm). From the transmittance measurements made in air (shown in Figure 1e), we can further infer that the shifts of peak maxima that occur as a result of changes made in the metal overlayer composition are small if nanohole geometries are identical. We therefore selected the p580 nanohole array as a suitable exemplar for the bulk RI sensitivity evaluation of bimetallic PCs carried out in this study.

**Figure 3:**
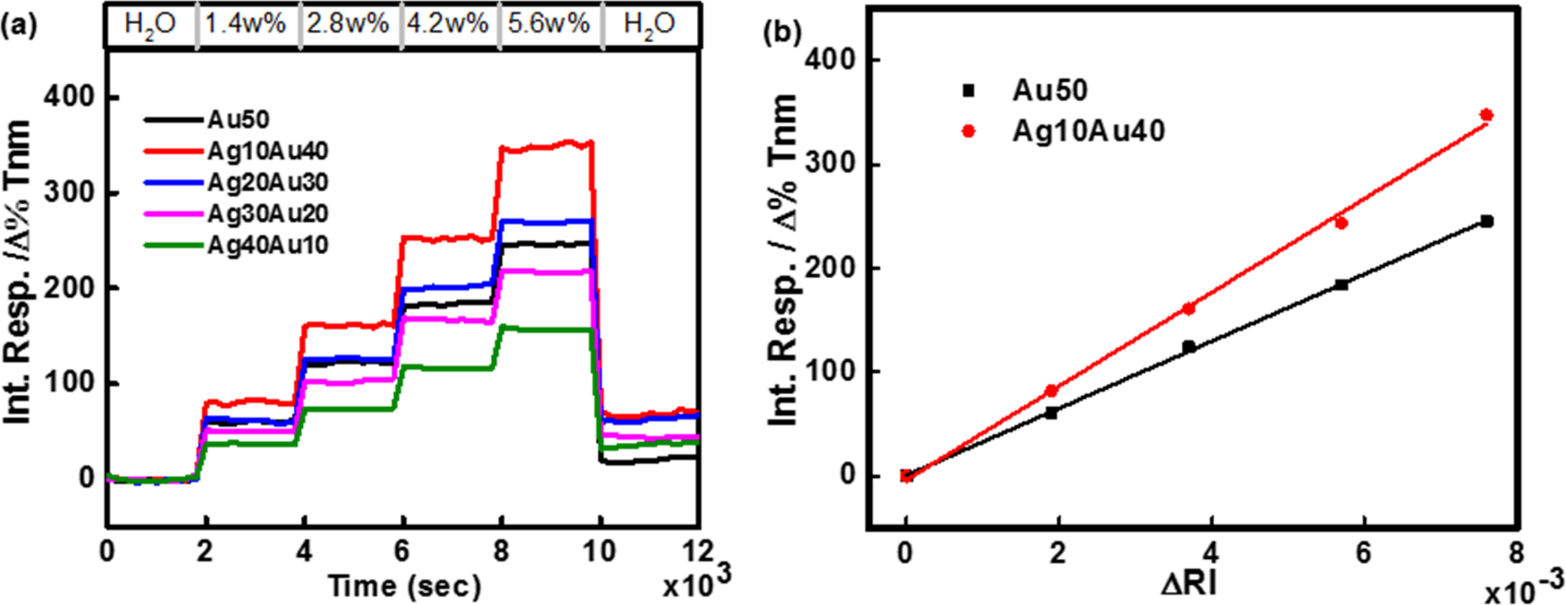
(a) Integrated multispectral response of five full-3D PCs with different metal composition to injections of increasing concentration of aqueous PEG solutions (from 1.4 to 5.6 wt %). Transmittance changes as a function of time were collected over a wavelength range of 355–1000 nm. (b) Linear change in the average integrated response with refractive index change on Au50 and Ag10Au40 full-3D PCs.

Figure 3a shows the integrated responses of five full-3D PCs selected for a design rule giving optimal RI responses with different metal compositions, here plotted as a function of time. The small baseline shift seen in the data over the course of the measurement is due to a small, uncompensated change in temperature. Figure 3b demonstrates the linear integrated response changes for the Au50 and Ag10Au40 full-3D PC as a consequence of changes made in the bulk refractive index (ΔRI) of the contacting PEG solutions.

The Ag10Au40 full-3D PC exhibits the largest RI sensitivity among the five PCs studied (the FOM values measured for these PCs are listed in Table 1). From 355–1000 nm, the RI sensitivity for the Ag10Au40 bimetallic PC (45,000 Δ%*T*·nm/RIU) is nearly ≈1.41 times that of the Au thin film PC (32,000 Δ%*T*·nm/RIU). Higher mass-fractions of Ag in the bilayer stack progressively weaken the plasmonic response, as seen in the lower values of the FOM.

**Table 1:** Figure-of-merit (FOM, Δ%*T*·nm/RIU) for PCs with different ratios of Ag and Au.

Wavelength (nm)	Au50	Ag10Au40	Ag20Au30	Ag30Au20	Ag40Au10

355–1000	32,000	45,000	36,000	29,000	21,000

Transmission spectra measured in water provide an important qualitative insight into the differences in the bulk RI sensitivity of the PCs. We note that sharp, intense optical features are more significantly weighted in the multispectral RI response calculation (Equation 1) as compared to broad, weak transmission features. Supporting Information File 1, Figure S3a shows the transmission spectra of PCs measured in water that illustrate these effects. Of particular note are the transmission intensity minima (appearing as sharp features near 600 nm and 800 nm) and peak transmission maxima (a generally more complex feature appearing above 800 nm). One sees here that the full-3D Ag10Au40 PCs, the best system for bulk RI sensing, has the narrowest peak minima and the highest transmission feature (seen here at ≈900 nm) as the qualitative associations described above predict. The FDTD results calculated for PCs immersed in water do not agree perfectly with the transmission spectra acquired experimentally because the mounted PDMS flow cell is not considered in the calculation. To allow solution flow and direct contact with liquids during measurements, a closed sampling cell is required and, in such cases, presents a glass–solution interface through which the incident radiation passes. Due to its complexity, the presence of this glass layer was not accounted for within the FDTD simulation; it is this omission to which the observed deviations are ascribed.

They do, however, still affirm the intuitive correlations noted above. Considering the calculated transmission spectra in water for the five PCs (shown in Supporting Information File 1, Figure S3b), as predicted, the Ag10Au40 full-3D PC presents both the narrowest peak minima and highest intensity in the calculated transmission response. Overall, due to the structure of the fabricated device, there will be an intrinsic sensitivity to certain variable physical parameters, such as the exact specifications of the flow cell geometry and/or the overall optical path length due to the mounting of the device in the radiation path. Ultimately, this will lead to slight variations in analytical sensitivity, requiring rigorous experimental design to provide integrated plasmonic devices with suitable analytical sensitivities for detection with high reproducibility.

### SERS enhancement of bimetallic plasmonic crystals

**Figure 4:**
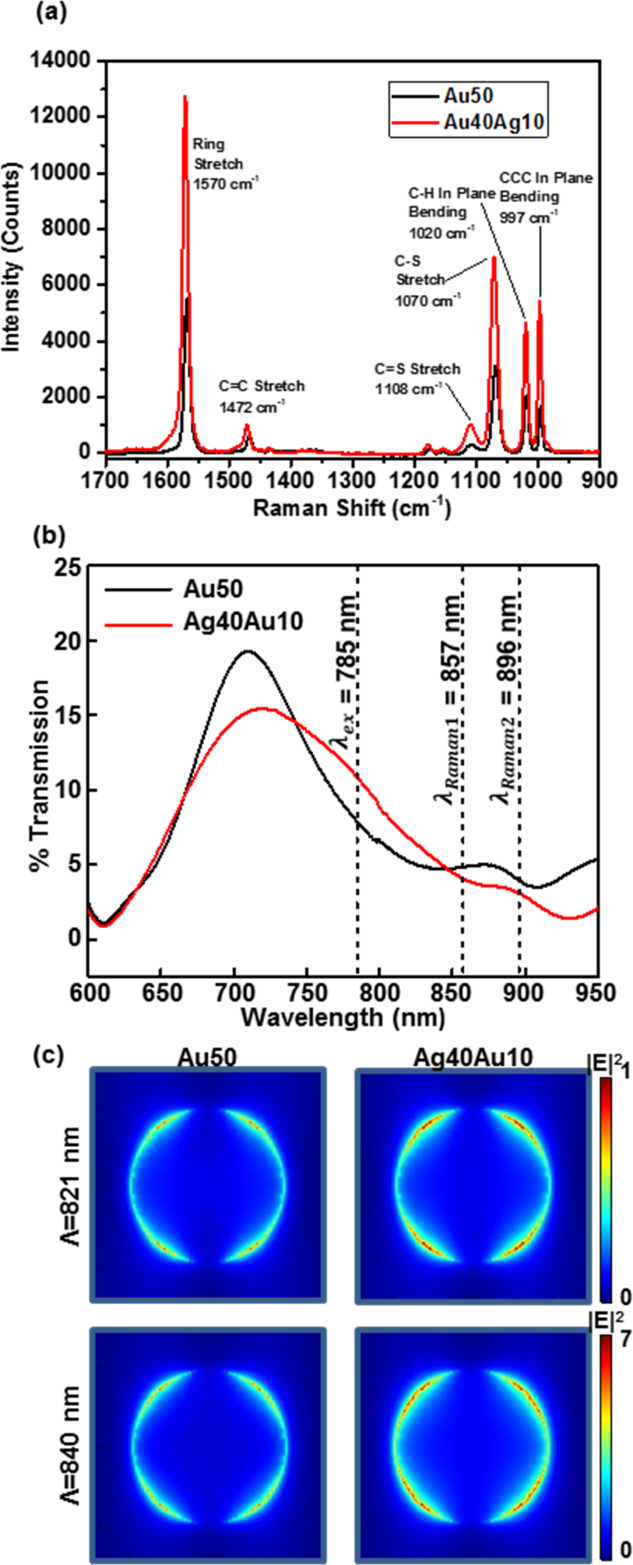
(a) SERS spectra of benzenethiol adsorbed onto Au50 and Ag40Au10 quasi-3D PCs measured with laser power of ≈5 mW. (b) Experimental transmission spectra in air for Au50 and Ag40Au10 quasi-3D PCs used to collect the Raman spectra in (a). The locations of the laser excitation and Raman peaks are marked as λ_ex_, λ_Raman1_ and λ_Raman2_, respectively. (c) FDTD-computed top (*x*–*y*) intensity distribution at 5 nm away from the surface of Au50 and Ag40Au10 quasi-3D PCs at λ = 821 nm and 840 nm.

Nanohole arrayed PCs are also promising as an easily replicable substrate for SERS. We have shown in an earlier report that plasmonic crystals formed on a molded SOG substrate provided excellent performance for Raman spectroscopy measurements [26]. In this study, we demonstrate that even greater Raman signal enhancements are achievable by using a bimetallic PC compared to those offered by more conventional monometallic systems. The total double metal layer thickness (50 nm) and the SOG nanohole array design rules (580 nm periodicity and 380 nm diameter) used for the SERS measurement are the same as those adopted in the RI measurements described above. Optimized thicknesses of the Ag and Au metal films on the embossed SOG PC were tested using the quasi-3D PC device motif, which was found to be a more suitable design for SERS measurement in earlier work [25]. The data presented in Supporting Information File 1, Figure S4 experimentally confirmed that a larger SERS enhancement does in fact result for the quasi-3D PC compared to the full-3D design for the bimetallic case as well. Table 2 shows the Raman intensities measured for each quasi-3D PC as a function of the different Au and Ag thickness ratios. As the data reveal, the best SERS enhancement is provided by the Ag40Au10 quasi-3D PC, with a Raman intensity that was ≈2.3 times higher than that of a comparable Au-coated PCs (shown in Figure 4).

**Table 2:** Raman intensity (counts) at 1073 cm^−1^ and 1573 cm^−1^ for five different full-3D PCs with 580 nm periodicity.

	Au50	Ag10Au40	Ag20Au30	Ag30Au20	Ag40Au10

1073 cm^−1^	3133	3395	4461	4845	7095
1573 cm^−1^	5499	5048	6437	7359	12830

The major Raman active vibrational modes of benzenethiol are located at 1073 cm^−1^ and 1574 cm^−1^ (which correspond to Raman shift peaks relative to the 785 nm laser excitation at 857 nm and 896 nm, respectively) [64]. Past work has noted that an approximate requirement for SERS enhancement by a PC is an enhanced electric field following at an optical frequency halfway between the laser excitation wavelength (λ_ex_ = 785 nm) and the scattered wavelength of the Raman active mode of interest [25,64–65]. This, then, corresponds to the case for the noted modes of benzenethiol where the PC provides maximal transmittance at wavelengths of 821 nm and 840 nm (the halfway point between λ_ex_ and λ_Raman_). The experimental transmission spectra of Au50 and Ag40Au10 quasi-3D PCs measured in air shown in Figure 4b qualitatively confirm this correlation between the magnitude of the SERS enhancement and the underlying optical properties of the plasmonic substrate.

FDTD simulations theoretically confirm the underlying mechanism and optimized PC structures for SERS enhancement. Calculated top field intensity distributions at 5 nm away from the surface of PCs (shown in Figure 4c) exhibit that the Ag40Au10 quasi-3D PC generates a stronger enhanced field compared with Au50 quasi-3D PC, which is in good accord with the correlation between the strength of the plasmonic resonance and the SERS intensity. SERS enhancement theory suggests that the intensity of the Raman scattered radiation is proportional to the square of the electric field [12]. FDTD-computed electromagnetic SERS enhancement factors (|*E*|^4^) for PCs with different Au layer thicknesses at wavelengths of 821 nm and 840 nm show similar trends as compared to the experimental results in that the SERS intensity increases as the thickness of the Ag layer increases (presented in Supporting Information File 1, Figure S5). The quantitative discrepancies between the measured SERS intensities and calculated enhancement factors as a function of the Au layer thickness likely originate as a consequence of the inexact model used for quasi-3D PCs (illustrated in Figure 1a). The latter does not take into account that there are some metal particles on the sidewall near the bottom disk and top layer (shown in inset of Figure 1c), which are structures that can contribute to the spectroscopic response of the PC optic.

These correlations are not fully predictive of optimal performance in SERS for a broader range of PC design rules. The larger data, for example, specifically show that the p580 PC is not the best design rule for SERS measurements and that the optical properties needed to obtain an optimized performance are more complex and likely application-specific. We have found that by using benzenethiol as an exemplary reporter molecule, for instance, signals of a p780 quasi-3D Ag40Au10 PC are higher than those of the p580 quasi-3D Ag40Au10 PC, demonstrating an approximately ≈1.3–1.8-fold enhancement (Supporting Information File 1, Figure S6a). The general correlation of the SERS intensity enhancement with specific features of the transmission intensity apparently still retain a useful qualitative character when one notes that the experimental results show higher transmission values at 820 nm and 841 nm for the p780 PC as compared with the p580 PC case (shown in Supporting Information File 1, Figure S6b). We believe further work, concomitant with theoretical insights, will be required to better understand associations between the optical properties and performance, as was observed here in this work.

## Conclusion

The influence of the mass coverage of Ag and Au thin films as well as the geometrical parameters of the nanohole arrays of bimetallic (bilayer) plasmonic crystals were characterized through multispectral bulk RI sensing and SERS measurements combined with insights from theoretical modeling. This work demonstrates the feasibility of Ag/Au bimetallic PCs for analytical applications, further establishing that bimetallic systems markedly surpass the quantitative performance of monometallic systems reported previously. With FDTD calculations, the nature of the different optical and plasmonic features that provide these performance attributes was confirmed. An optimized system was developed through careful engineering of the system and analysis of the transmission spectra both experimentally and computationally. The methods described here hold additional value in that they provide guidance for further optimization of a design framework for plasmonic devices to suit specific features of analytical applications. More directly, though, the multilayered bimetallic PC is found to offer great flexibility in terms of ease of fabrication and features of performance, thereby establishing it as an interesting platform for highly sensitive forms of plasmonic-based chemical sensing.

## Supporting Information

File 1 Additional theoretical and experimental information.
Details: Theoretical analysis of Bloch wave surface plasmon polaritons and Wood’s anomalies of PCs; Schematic illustration of a nanohole; Experimental and FDTD calculated transmission spectra in water of the full-3D PCs; Integrated multispectral response and computational normal incident transmission spectra of full-3D PCs with different nanohole array periodicity; SERS spectra of benzenethiol and experimental transmission spectra in air for Ag40Au10 quasi-3D PC with 580 nm and 780 nm periodicity; SERS spectra of benzenethiol adsorbed onto full-3D and quasi-3D PCs.
