# Supplementary material for: Refractive index sensing and surface-enhanced Raman spectroscopy using silver–gold layered bimetallic plasmonic crystals
Source: Beilstein J Nanotechnol. 2017 Nov 24;8:2492–503. doi: 10.3762/bjnano.8.249 (PMC5704757; doi:10.3762/bjnano.8.249)
Supplement: File 1 — Additional theoretical and experimental information. Details: Theoretical analysis of Bloch wave surface plasmon polaritons and Wood’s anomalies of PCs; Schematic illustration of a nanohole; Experimental and FDTD calculated transmission spectra in water of the full-3D PCs; Integrated multispectral response and computational normal incident transmission spectra of full-3D PCs with different nanohole array periodicity; SERS spectra of benzenethiol and experimental transmission spectra in air for Ag40Au10 quasi-3D PC with 580 nm and 780 nm periodicity; SERS spectra of benzenethiol adsorbed onto full-3D and quasi-3D PCs. [file Beilstein_J_Nanotechnol-08-2492-s001.pdf]

Supporting Information

for

Refractive index sensing and surface-enhanced Raman spectroscopy using silver–gold layered bimetallic plasmonic crystals

Somi Kang[1], Sean E. Lehman[2], Matthew V. Schulmerich[3], An-Phong Le[2], Tae-woo Lee[4], Stephen K. Gray[4], Rohit Bhargava[3], and Ralph G. Nuzzo[*1,2]

Address: [1]Department of Materials Science and Engineering, University of Illinois at Urbana-Champaign, Urbana, Illinois 61801, USA, [2]Department of Chemistry, University of Illinois at Urbana-Champaign, Urbana, Illinois 61801, USA, [3]Beckman Institute for Advanced Science and Technology, University of Illinois at Urbana-Champaign, Urbana, Illinois 61801, USA, and [4]Chemistry Division and Center for Nanoscale Materials, Argonne National Laboratory, 9700 Cass Ave., Lemont, Illinois 60439, USA

Email: Ralph G. Nuzzo - r-nuzzo@illinois.edu

*Corresponding author

Additional Theoretical and Experimental Information

Theoretical Analysis of Bloch Wave Surface Plasmon Polaritons (BW-SPPs) and Wood's Anomalies (WAs) of Plasmonic Crystals

BW-SPPs are standing waves corresponding to the coherent superposition of propagating SPPs [1,2]. An approximate relation for the allowed wavelength of BW-SPPs on a nanohole array is

$$\lambda \approx \frac{P}{\sqrt{n_x^2 + n_y^2}} \sqrt{\frac{\varepsilon_{metal}(\lambda)\varepsilon_d}{\varepsilon_{metal}(\lambda) + \varepsilon_d}} \tag{S1}$$

where P is the nanohole lattice spacing, $\varepsilon_{metal}(\lambda)$ is the wavelength dependent relative dielectric constant for metal, and ε_d is the relative dielectric constant of the dielectric material that interfaces with the metal film [3]. BW-SPPs of bimetallic plasmonic crystal are generated at the air/gold interfaces ($\varepsilon_d = 1$ for air) and the silver/SOG interfaces ($\varepsilon_d = 1.43$ for SOG).

TABLE S1. Zero-order wavelength of the BW-SPPs (unit of wavelength is nm)

n_x	n_y	air/Au	Au/SOG	air/Ag	Ag/SOG
0	1	611	860	602	857
0	2	331	303<br>415	346<br>370	308<br>353<br>484
1	1	415	642	449	629
1	2	316	305<br>384	-	356<br>453
2	2	-	310<br>346	-	361<br>410

The solutions of Eq. S1 excluding $n_x = n_y = 0$ are shown in Table S1, which yield the discrete zero-order wavelength of the BW-SPPs. Table S1 also includes solutions for BW-SPPs wavelength for gold/SOG interfaces and air/silver interfaces for mono-layered plasmonic crystals (Au50 and Ag50).

The Wood's anomalies (WAs) are light waves diffracted to propagate in the plane of the surface [4-6]. WAs act in the same fashion as BW-SPPs, but have a very narrow spatially extended nature without direct involvement of plasmonic responses. The condition for WAs is

$$\lambda = P\frac{\sqrt{\varepsilon_d}}{\sqrt{n_x^2 + n_y^2}} \tag{S2}$$

Table S2 shows the wavelength of WAs at air/metal and metal/SOG interfaces.

TABLE S2. Wavelength of the BW-SPPs (unit of wavelength is nm)

n_x	n_y	air/metal	metal/SOG
0	1	580.0000	693.5791
0	2	290.0000	346.7896
1	1	410.1219	490.4345
1	2	259.3839	310.1780
2	2	205.0609	245.2173

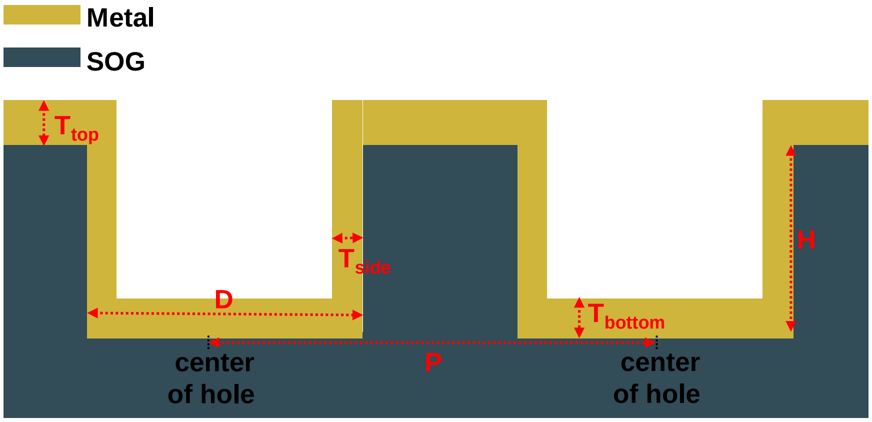

Figure S1. Schematic illustration of an individual nanohole on a full 3D plasmonic crystal used for FDTD modeling. For simplicity, we assumed that metal films are uniformly distributed on a cylindrical SOG nanohole with a right angle edge. The full 3D plasmonic crystal with the periodicity (P) of hole of 580 nm, hole depth (H) of 340 nm, and diameter of hole (D) of 380 nm are used for all FDTD calculation. The sidewall thickness (T_{side}) for Ag and Au mono-metallic plasmonic crystals used in these calculations were slightly different (16 nm for Ag sidewall and 12 nm for Au sidewall), while thicknesses of top and bottom layers (T_{top}=50 nm and T_{bottom}= 20 nm) for both plasmonic metal layer were same. We only monitored top layer thickness of metal to control thickness of metallic resonant layer without consideration of sidewall thickness.

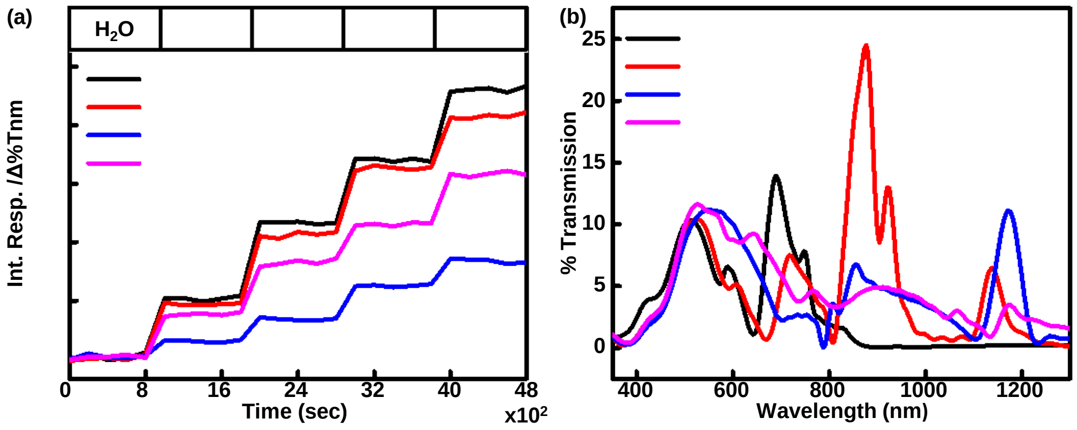

Figure S2. (a) Integrated multispectral responses of full 3D plasmonic crystals with different periodicity of nanohole arrays (~ 580 nm, ~780 nm, ~1100 nm, and ~1600 nm periodicities) to injections of increasing concentration of aqueous PEG solutions (from 1.4 w% to 5.6 w%). Transmittance changes as a function of time were collected over a wavelength range of 300-800 nm. (b) FDTD-calculated normal incident transmission spectra of full 3D plasmonic crystals with different periodicity of nanohole arrays was used in bulk refractive index sensing in (a).

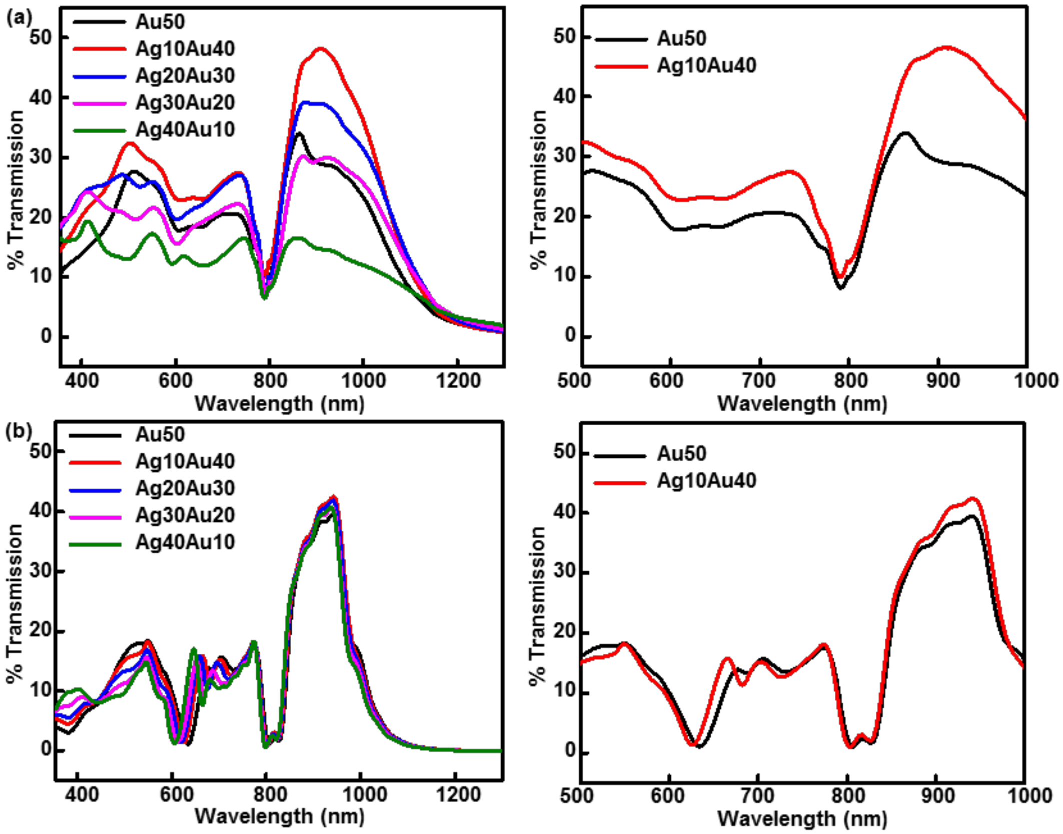

Figure S3. (a) Left: experimental transmission spectra in water of five full 3D plasmonic crystals with different metal compositions. Right: Magnified view of the transmission spectra of Au50 and Ag10Au40 full 3D plasmonic crystals over a wavelength range of 500-1000 nm. (b) FDTD calculated transmission spectra in water of the full 3D plasmonic crystals with five different metal compositions. Right: Magnified view of the transmission spectra of Au50 and Ag10Au40 full 3D plasmonic crystals over a wavelength range of 500-1000 nm.

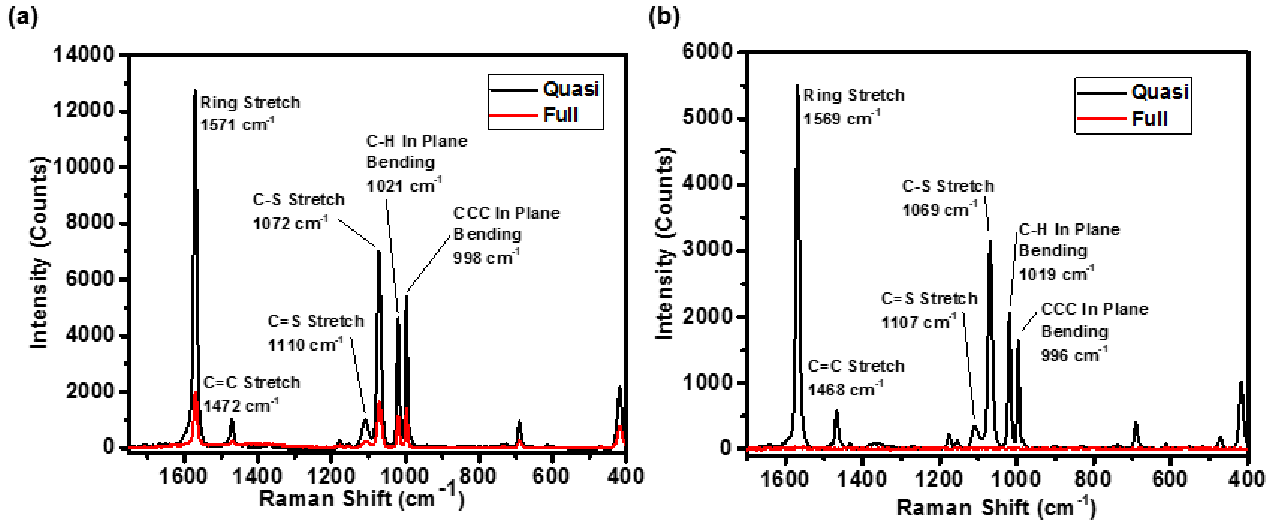

Figure S4. (a) SERS spectra of benzenethiol adsorbed onto full 3D and quasi 3D Ag40Au10 plasmonic crystals measured with laser power of ~5 mW. (b) SERS spectra of benzenethiol adsorbed onto full 3D and quasi 3D Au50 plasmonic crystals measured with laser power ~5 mW.

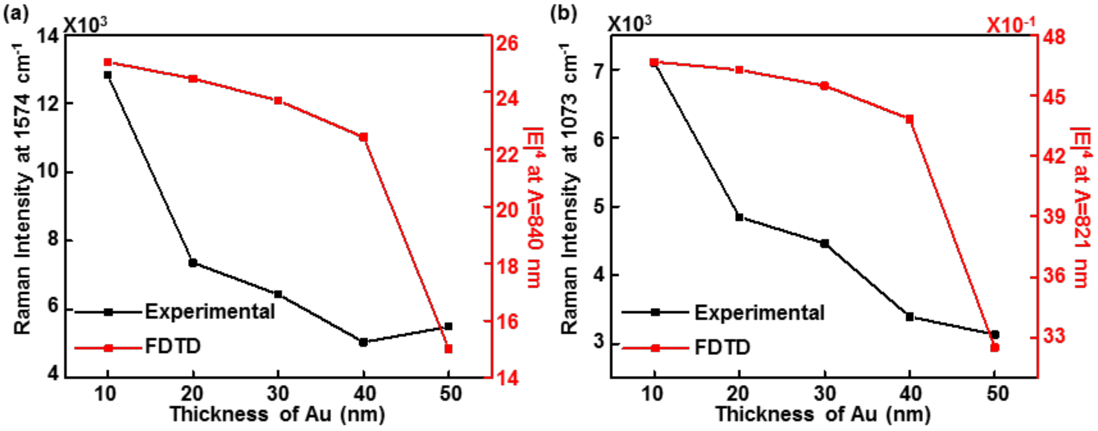

Figure S5. Measured variation of the Raman intensity as a function of thickness of Au and corresponding FDTD-computed average of squared field intensity ($|E|^4$) at 4 nm away from the surfaces of plasmonic crystal for (a) $\lambda_{Raman2} = 1574cm^{-1}$ and (b) $\lambda_{Raman1} = 1073cm^{-1}$.

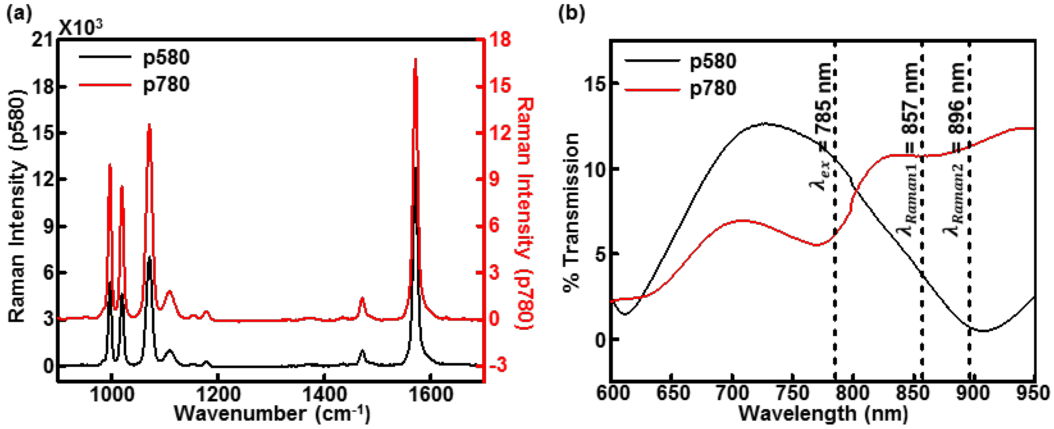

Figure S6. (a) SERS spectra of benzenethiol adsorbed onto Ag40Au10 quasi 3D plasmonic crystals with ~580 nm and ~780 nm of periodicity measured with laser power of ~5 mW. (b) Experimental transmission spectra in air for Ag40Au10 quasi 3D plasmonic crystals with ~580 nm and ~780 nm periodicity used to collect the Raman spectra in (a). The locations of the laser excitation and Raman peaks are marked as λ_{ex}, λ_{Raman1}, and λ_{Raman2}, respectively.

REFERENCES

(1) Salomon, L.; Grillot, F.; Zayats, A. V.; de Fornel, F. *Phys. Rev. Lett.* **2001,** *86* (6), 1110-1113.

(2) Darmanyan, S. A.; Zayats, A. V. *Phys. Rev. B* **2003,** *67* (3).

(3) Chang, S. H.; Gray, S. K.; Schatz, G. C. *Opt. Express* **2005,** *13* (8), 3150-3165.

(4) Treacy, M. M. J. *Appl. Phys. Lett.* **1999,** *75* (5), 606-608.

(5) Sarrazin, M.; Vigneron, J. P.; Vigoureux, J. M. *Phys. Rev. B* **2003,** *67* (8).

(6) Lezec, H. J.; Thio, T. *Opt. Express* **2004,** *12* (16), 3629-3651.
